# What Are Little Boys and Girls Made of? The Origins of Sexual Dimorphism

**DOI:** 10.1371/journal.pbio.1001905

**Published:** 2014-07-08

**Authors:** Roland G. Roberts

**Affiliations:** Public Library of Science, Cambridge, United Kingdom

Snips and snails and puppy dogs' tails, or sugar and spice and all things nice? From our animal-centered point of view, the distinction between males and female can seem rather obvious—many animals have male individuals and female individuals whose differences are often pretty evident at a glance. However, we're also aware of more exotic variations like hermaphroditism and Tiresias-like midlife gender-bending. Indeed, the further we get from the birds and the bees, the more it becomes unclear what exactly we mean by “male” and “female.” Are these just tags, or are they fundamentally different? And if the latter, how did this fundamental difference come to be?

Sex arose as a way of allowing the mixing and shuffling of genotypes between two individuals, generating a blended range of genotypes in the offspring that might maximize the chances of thriving in a changing environment. However, to get the most out of this you need to discourage mating with your nearest and dearest. Many unicellular organisms have solved this problem by evolving a mating type system—this typically involves a genetic switch that determines which of two or more optional “mating types” a given individual belongs to. It's essentially a compatibility check—mating can only occur between individuals of different mating type.

As life becomes more complex, however, so does sex. Multicellular organisms can have cells whose shape and function are tailored to a particular task, and can afford to delegate sex to a specialized subset of their cells—the gametes. But because the sexually active cells no longer have to perform as an independent unicellular organism, they're free to be influenced by complex evolutionary forces that further specialize them. So rather than remaining equal, a recurrent pattern emerges in gametes across the tree of life. The gametes of one mating type become small, mobile, and numerous to enable mating at a distance. And the gametes of the other mating type become large, immobile, and few to allow for effective provisioning of the developing multicellular offspring. This state of having sexually dimorphic gametes is called anisogamy (as opposed to isogamy), and the mating types are now true sexes—male and female respectively.

But what's the mechanism behind this striking phenomenon? Theorists have suggested that it could happen by the insertion of a gene that determines cell size next to the gene that determines mating type, but this hasn't been shown experimentally. Sa Geng, Peter DeHoff, and James Umen, the authors of a paper just published in *PLOS Biology*, have exploited two species of volvocine algae to probe how anisogamy arises and to test this theory. These two species, separated by 200 million years of evolution, couldn't be more different in their appearance or sexual proclivities. *Chlamydomonas reinhardtii* is a 10-µm single-celled organism with two flagella that mates isogamously with two mating types, “*plus*” and “*minus*.” *Volvox carteri* is a much larger multicellular ball comprising thousands of cells, and mates anisogamously with clearly differentiated sperm and egg borne by obviously dimorphic sexes, male and female.

**Figure 1:**
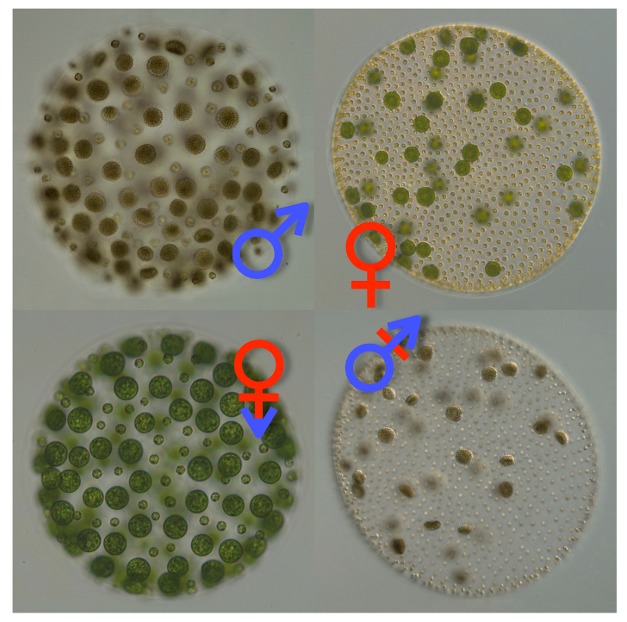
Wild-type and gender-swapped *Volvox*. Color differential interference contrast (DIC) images of *V. carteri* wild-type male (upper left), wild-type female (upper right), pseudo-female (lower left) and pseudo-male (lower right). *Image credit: Sa Geng and James Umen.*

Mating type in *Chlamydomonas* is already known to be determined genetically by the presence or absence of a gene called *CrMID*. This gene encodes a transcription factor that triggers the *minus* program and represses the *plus* one in response to a drop in nutrients in the environment. The sexual development of *Volvox*, on the other hand, is triggered by pheromones that are released by males but act on both sexes. This turns vegetative individuals (about 2,000 somatic cells and 16 reproductive “gonidial” cells) into reproductive individuals with pronounced sexual dimorphism; while the females contain up to 48 large egg cells set among about 2,000 somatic cells (Figure 1, upper right), the males contain only 128 somatic cells surrounded by 128 androgonidial ones (Figure 1, upper left). However, these male gonidia can *each* divide further to produce 128 sperm which swim as a packet to a female, enter her, and then swim individually to find an egg.

Like *Chlamydomonas*, mating type in *Volvox* is determined genetically, and the *Volvox* sex-determining locus has been mapped to a region containing a version of the *CrMID* gene, *VcMID*, which is only present in males. It seemed a fair assumption that *VcMID*, like its *Chlamydomonas* counterpart, would play a role in determining which sex the organisms were, but was it also responsible for driving the dimorphism?

To test this, the authors tried some “gender reassignment”—they first expressed fluorescently tagged VcMID protein inappropriately in genetically female *Volvox* and then triggered sexual development with pheromones. The result was an unusual pseudo-male state that, like females, had up to 48 gonidial cells surrounded by 2,000 somatic cells, but *then*, like males, each of those gonidial cells that would have normally become eggs divided further to make packets of rather aberrant sperm that were capable of fertilizing other females (Figure 1, lower right). They were also able to use the tagged protein to show that the production and nuclear localization of VcMID are regulated in a complex cell-type–specific manner.

But what happens if you do the reciprocal experiment; that is, to interfere with *VcMID* expression in male cells? To do this they used RNA interference (RNAi), a method that has been widely used to reduce levels of expression of specific genes in many organisms. However this was a first for *Volvox*, and the authors showed that they could use hairpin-shaped RNAs to specifically zap *VcMID* expression in males. Like the *VcMID*-expressing females, the *VcMID*-lite males entered into an odd sexual grey area. This time, though, they saw a pseudo-female state that initially had the normal male pattern of 128 somatic cells and 128 gonidial ones, but the latter failed to divide further into sperm (Figure 1, lower left). Indeed, these large gonidia could be fertilized, like normal eggs, by sperm from another male. Unlike the crosses formed between pseudo-male sperm and normal eggs, the progeny of pseudo-female eggs and normal sperm fared rather badly, with fewer than half surviving, so perhaps fully viable egg formation needs more than mere absence of VcMID.

Remarkably, the authors were able to show that this male/female switch is tunable—when they used a weaker version of the *VcMID* RNAi hairpin, the partial depletion of VcMID resulted in hermaphrodites that contained both pseudo-male sperm packets and pseudo-female eggs. And yes, they appeared to be self-fertile.

The authors conclude that *VcMID* not only determines mating type, like its *CrMID*-like ancestor, but that it also itself orchestrates many aspects of sexual dimorphism. The possibility arises that rather than anisogamy evolving from the recruitment of other genes to the sex-determining locus, as formerly proposed, it can evolve through changes that are intrinsic to the *MID* gene product. VcMID and CrMID, while being clearly descended from a common ancestor, have diverged considerably, and the authors, in fact, show that neither CrMID nor two different CrMID-VcMID chimeras can stand in for VcMID and drive pseudo-male development in *Volvox*.

This study seems to show that sexual dimorphism can arise from isogamy largely via adaptations of the sex-determining gene itself. These changes presumably re-wire its regulatory network, such that in an isogamous organism the output is simple mating type, while in an anisogamous one further, more complex developmental programs are initiated. And *that's* what little boys and girls are made of.

**Geng S, DeHoff P, Umen, JG (2014) Evolution of Sexes from an Ancestral Mating-Type Specification Pathway.** doi:10.1371/journal.pbio.1001904

